# Progress under the Mental Deficiency Act

**Published:** 1913-11-22

**Authors:** 


### PROGRESS UNDER THE MENTAL DEFICIENCY ACT.

We have recently directed attention to the work which lies before the Board of Control set up by the Mental Deficiency Act, but the march of events has brought new aspects of the subject into view. More important than the personnel of the Board is the constitution of the statutory committees. A certain amount of confusion has already appeared in connection with the relations of these committees with the pre-existing Asylums Committees of various local authorities. Under the Act some latitude in this respect is allowed; and the new committees can be formed from the old ones, or independently of them, at discretion. In London it was also suggested that the work hitherto done by the Asylums Committee should be transferred to a new body consisting partly of members of the Council and partly of persons (including women) appointed from outside the Council, the members of the Council to form a majority of the committee. This proposal was strenuously resisted by the members of the Asylums Committee, but was eventually carried in spite of their protests.

Further testimony of the difficulties which confront the authorities to whom the administration of the Act is confided is afforded by a very able letter to the *Times* from the pen of Dr. Robert Jones, of Claybury. He points out that the Act permits county and borough authorities to combine together to provide joint institutions. The advantage of such combination is that "homes" large enough to permit of proper classification of the varying degrees of mental deficiency can thus be created. Another suggestion is that county and borough Asylum Committees should provide the necessary accommodation in homes or detached villas adjoining their asylums. Thus those who became certifiably insane could be transferred at once into the asylum adjoining their home or villa.

Dr. Jones points out that the finance of the Act will allow of the treatment of only about 12,000 out of 66,000 mentally defective who really need it, and pleads for a conference summoned at the instance of a Department of State (presumably this would be the Home Office), or a delegation from the authorities empowered to carry out the provisions of the Act. Such a conference would strengthen the hands of the Board of Control, and by bringing administrators and their critics together would be of inestimable advantage to the public purse. The public does not realise, says Dr. Jones, that to house and treat the whole of the weak-minded in separate institutions will cost millions. We agree with this expert that a close association of the working of the Mental Deficiency Act with that of the Lunacy Acts is not only the most economical plan, but also by far the best in the interests of the patients themselves.

